# Hardening of Nanoporous Au Induced by Exposure to Different Gaseous Environments

**DOI:** 10.3390/ma15082718

**Published:** 2022-04-07

**Authors:** Giorgio Pia, Elisa Sogne, Andrea Falqui, Francesco Delogu

**Affiliations:** 1 Dipartimento di Ingegneria Meccanica, Chimica e dei Materiali, Università Degli Studi di Cagliari, Piazza d’Armi, 09123 Cagliari, Italy; francesco.delogu@unica.it; 2 NABLA Lab, Biological and Environmental Sciences and Engineering (BESE) Division, King Abdullah University of Science and Technology (KAUST), Thuwal 23955-6900, Saudi Arabia; eli.sogne@gmail.com; 3 Dipartimento di Fisica “Aldo Pontremoli”, Università Degli Studi di Milano, Via Celoria 16, 20133 Milan, Italy; andrea.falqui@unimi.it

**Keywords:** metals and alloys, porous materials, nanoporous Au, coarsening, kinetics

## Abstract

This work focuses on the mechanical behaviour of nanoporous Au samples alternately exposed to ozone and carbon dioxide. Nanoporous Au was fabricated by freely corroding the Ag_70_Au_30_ parent alloys prepared by mechanical alloying in the form of powder and subsequently compacted by cold pressing. Dealloying was performed in acidic solution, and conditions were suitably adjusted to obtain fine nanoporous Au structures with ligaments about 15 nm thick. Nanoporous Au samples with increasingly thicker ligaments, up to about 40 nm, were fabricated by annealing the pristine nanoporous Au structure for different time intervals at 473 K. For all of the samples, the cyclic variation of gaseous atmosphere results in a macroscopic strain variation due to the occurrence of surface oxidation and reduction processes. We show that the reiterated cyclic exposure to the different gases also induces the progressive hardening of nanoporous Au, which can be ascribed to irreversible strain contributions. For nanoporous Au samples with ligaments that are 15 nm thick, after 50 exposure cycles, the yield strength increases approximately from 49 MPa to 57 MPa. A systematic investigation on coarser nanoporous Au structures indicates that, with the same exposure cycles, the degree of hardening decreases with the ligament thickness.

## 1. Introduction

Monolithic nanoporous (NP) metal foams constitute a broad class of materials with unique structure and properties [1]. The structure resembles a disordered three-dimensional maze of condensed open cells where relatively thick ligaments interconnect with massive nodes. Voids and matter are very close to each other on the nanometre scale, giving rise to two bi-continuous, complementary networks [1]. The intricate architecture is the result of the atomic-scale selective dissolution of less noble chemical elements from Au-based binary or multinary alloys [1]. Proceeding gradually via the removal of individual atoms from the alloy surface and the subsequent rearrangement of neighbouring sites, dealloying eventually induces deep changes in the surface morphologies and the local degree of porosity [1]. As corrosion pits deepen, forming irregularly oriented elongated cavities, their interconnection becomes more and more probable and, finally, a network of channels develops [1]. The resulting structure consists of solid ligaments and nodes that have characteristic lengths reduced down to the nanometre size, which imparts on the material intriguing physical and chemical properties that, presently, make NP metals one of the most intensively studied subjects in materials science [1,2,3,4,5].

NP Au foams provide the ideal case study for the entire class of NP metals [6]. On the one hand, an experimental investigation on NP Au has clearly shown that NP metals can exhibit highly specific physical and chemical properties that do not find correspondence in their massive counterparts. On the other hand, NP Au has its own significance for various application. In this respect, sensing, catalysis and structural engineering represent fields that already benefit, or can potentially benefit, from NP Au foams [7,8,9,10].

Together with the irregular architecture and morphologies, the metallic nature of chemical bonding makes NP Au foams exhibit remarkable catalytic activity in CO oxidation processes at low temperatures [8,11,12]. Moreover, the high surface-area-to-volume ratio of NP Au has been exploited to perform efficient and reversible liquid phase imbibition processes that can be controlled using suitable electric fields to finely tune the tension at solid–liquid interfaces [13].

The sensitivity of NP Au foams to chemical surroundings also renders them capable of mechanical responses [14,15]. In this regard, changes in the gaseous environment can make NP Au undergo reversible strain. In particular, literature indicates that NP Au structures with 20 nm thick ligaments can exhibit elastic strain with amplitude up to 0.5% when exposed alternately to a mixture of 7% ozone (O_3_) in oxygen (O_2_) and pure carbon monoxide (CO) [16]. Similarly, NP Au foams exposed to air with relative humidity varying cyclically in the range between 36% and 53% exhibit elastic strain amplitudes that can reach values of about 0.02% [17].

The reversible strain variation undergone by NP Au foams can be ascribed to the occurrence of absorption processes of gaseous species on the NP Au free surfaces. In turn, absorption induces the variation in intrinsic surface stresses that arises as a consequence of the fact that characteristic lengths are found on the nanometre scale [16,17]. Specifically, chemical interactions between the surface and gas generate relatively intense shear components along the ligament axis, which finally results in anisotropic local strains heterogeneously distributed across the NP Au foam [16,17].

Concerning the absorption-induced strain behaviour, more recent work has shown that irreversible strain contributions can also emerge during the cyclic exposure of NP Au to O_3_ in O_2_ and CO gaseous environments [18]. Indeed, NP Au structures with ligaments 15 nm thick showed a yield strength enhanced by 20% after 150 exposure cycles [18].

This work deepens insight into the strain-hardening behaviour of NP Au foams. To such aim, we fabricated NP Au structures with ligaments of different sizes. To this aim, we performed the controlled coarsening of NP Au structures with ligaments 15 nm thick obtained by dealloying. After performing the thermal annealing, we subjected the initial NP Au foams and the coarsened samples to cycles of alternate exposure to O_3_ in O_2_ and CO atmospheres. The experiments clearly show that the exposure cycles induce the strain hardening of NP Au foams and that the average ligament thickness definitely affects the strengthening behaviour.

The emerging relationship between hardening rate and ligament size is not anticipated by any theoretical approach or experimental evidence developed or published so far. It poses intriguing questions on the elasticity at the nanometre scale as well as on the role of surface chemistry in controlling actuation via NP structures. In the following, we provide the necessary details to reproduce our experiments and a detailed discussion of the experimental evidence obtained.

## 2. Materials and Methods

NP Au foams were fabricated by chemical dealloying of a bulk Ag_70_Au_30_ alloy. The Ag_70_Au_30_ alloy was obtained in the form of powder by mechanical alloying of elemental metals in a SPEX Mixer/Mill 8000 [19]. Afterwards, the alloy powders were annealed at 523 K for 10 h under Ar flux conditions in a tubular quartz reactor placed inside a Nabertherm N60/ER furnace and, then, compacted into cylindrical pellets about 5 mm in diameter and 0.2 mm thick using a cold-pressing device [19]. Subsequently, the cylindrical pellets were subjected to chemical dealloying. With this aim, they were plunged for 8 h in an aqueous solution of nitric acid at 70% at room temperature [19]. Nitric acid induced the selective dissolution of Ag and the consequent formation of NP Au foams with average ligament thickness and relative density approximately equal to 15 nm and 0.294, respectively. Chemical analysis performed by X-ray fluorescence revealed a residual Ag content of about 1 at%.

Coarsening was induced by annealing the NP Au foams at 473 K for selected time intervals between 0 and 5 h under Ar flux conditions. Annealing was performed in a parallelepiped-shaped quartz reactor placed inside a Nabertherm N60/ER furnace kept at 473 K. This temperature is only slightly higher than the minimum temperature of about 458 K reported to be needed to activate coarsening in NP Au foams [20]. Therefore, it allows for performing coarsening processes slow enough to enable fine control of the resulting NP Au structure.

The structure of NP Au foams was studied by scanning and transmission electron microscopy (SEM and TEM, respectively). SEM and TEM observations were performed using a Zeiss EVO LS15 SEM microscope and a FEI Tecnai G12 TEM microscope. Average characteristic lengths of NP Au structures were estimated performing measurements over 50 different ligaments and nodes.

Elongation and compression of NP Au samples in response to gaseous atmosphere were measured inside a glass compartment. The NP Au sample was fixed on a stable support and exposed to the desired chemical environment. Based on literature indications [16,18], NP Au was preliminarily heated up to 353 K for 5 min in a nitrogen (N_2_) flux to desorb and remove chemical species possibly adsorbed on the surface. Afterwards, the NP Au foam was exposed first to a gaseous mixture of 7% O_3_ in O_2_ for 30 min and, then, to pure CO for 25 min. Each change of gaseous flow was preceded by exposition to N_2_ for 2.5 min. As a consequence, each exposure cycle, performed at 298 K, consisted of four consecutive stages. The selected NP Au structures were subjected to a maximum of 50 exposure cycles.

The strain affecting NP Au foams exposed to gaseous environments was measured in situ using an Orton 2010 B dilatometer equipped with a LVDT sensor with a 20 nm resolution.

The mechanical properties of the NP Au samples subjected to cyclic exposure were measured ex situ using depth-sensing nanoindentation. Measurements were performed at 298 K by a Nano Test Vantage Micro Materials indenter. A calibrated three-sided pyramid diamond Berkovich tip with radius of about 200 nm was used. NP Au samples were loaded at the constant rate of 500 N s^−1^ using indentation loads between 400 and 2400 N. Correspondingly, indent depths ranged approximately from 200 to 800 nm. Yield strength was estimated analysing loading and unloading curves. Specifically, NP Au hardness was calculated dividing the maximum load by the indentation area observed after the Berkovich tip removal. The final hardness value is the average over 30 indents per pellet. The literature suggests that the yield strength of NP Au foams with relative densities of 0.3 or less is approximately equal to hardness [21,22]. Following the literature, we used a yield strength equal to hardness.

## 3. Results

**Figure 1:**
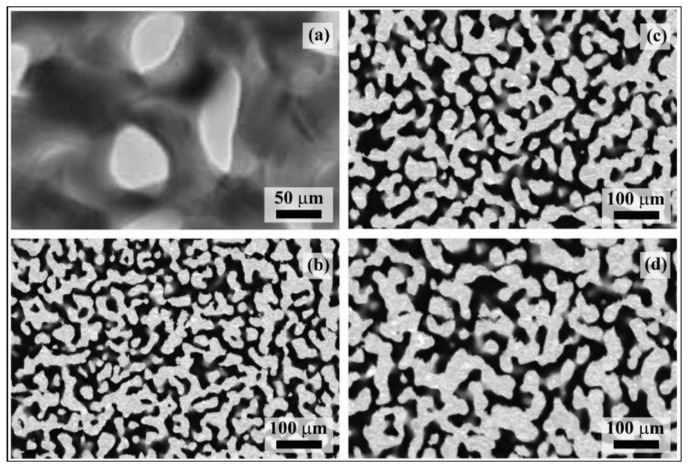
(**a**) TEM and (**b**) SEM micrographs of the NP Au foam obtained by dealloying. SEM micrographs refer to NP Au structures annealed for (**c**) 2 h and (**d**) 5 h.

The TEM and SEM micrographs reported, respectively, in Figure 1a,b show that dealloying determines the formation of a fine NP Au structure with ligaments approximately 15 nm thick. Ligaments have irregular shape and interconnect with each other at nodes that are relatively massive compared with ligaments. The fine structure of pristine NP Au samples is thermodynamically unstable. In particular, a significant driving force exists that pushes towards the minimization of Gibbs free energy via a reduction in the free surface area, with surface energy being a positive contribution to the overall Gibbs free energy of the structure.

Isothermal annealing at 473 K is sufficient to induce progressive coarsening of the NP Au structure. Accordingly, the SEM micrographs in Figure 1c,d reveal gradual increases in ligament and node size. Based on the literature reports [23,24,25,26], coarsening can be expected to take place via surface diffusion processes driven by the thermodynamic tendency to minimize Gibbs free energy. Our data support such a mechanistic scenario.

**Figure 2:**
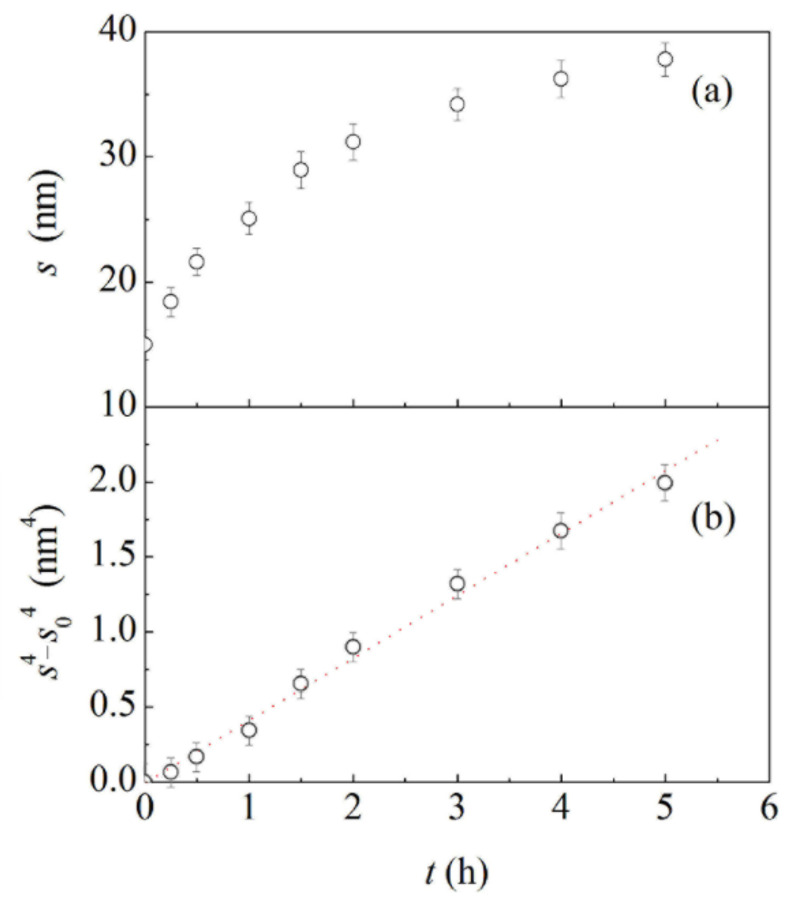
The average ligament thickness, s, and (**b**) the quantity, $s^{4}-s_{0}^{4}$, as a function of annealing time, t. Best-fitted line is also shown.

In this regard, relevant data are shown in Figure 2a, where the average ligament thickness, s, is plotted as a function of annealing time, t. Starting from an initial value, $s_{0}$, s increases according to a monotonic trend characterized by a gradual reduction in the variation rate. Coarsening kinetics can be better appreciated from Figure 2b, where s4−s0 is plotted as a function of t. In agreement with the literature [20,27,28,29], the plot is approximately linear, which strongly suggests a primary role of surface diffusion in the thickening of structural elements.

The availability of NP Au structures with different ligament sizes paves the way toward characterization of their mechanical response upon cyclic exposure to different atmospheres. In this respect, it is worth noting that the ligament size has been intentionally kept within the 15–40 nm range. Within this range, indeed, coarsening does not involve any detectable change of the overall density of the NP Au samples. Indirect measurements based on SEM and TEM image analysis invariably result in a solid fraction of about 0.297. Therefore, any difference in the mechanical response of different NP Au structures can be exclusively ascribed to the change of ligament size consequent to coarsening.

**Figure 3:**
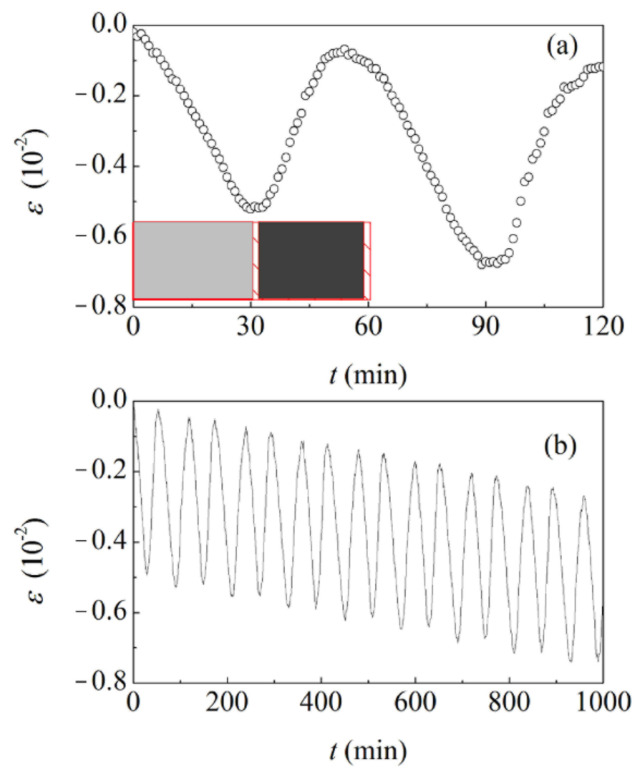
(**a**) The strain, *ε*, as a function of time, *t*, under cyclic exposure conditions. Exposures to O_3_ in O_2_ (light gray), N_2_ (shaded area), and CO (dark gray) are indicated for the first cycle. (**b**) The strain, as a function of the number of exposure cycles, *n*. Data refer to the NP Au foam obtained by dealloying.

The cyclic exposure of initial and coarsened NP Au foams to the selected gaseous environments induces an evident detectable strain response. Data concerning the initial NP Au foam with ligaments about 15 nm thick are shown in Figure 3a. As already observed in previous work [16,17,18], the NP Au structure shrinks in the presence of O_3_. Shrinkage is volumetric, and no detectable anisotropy is observed.

The observed behaviour can be related to the occurrence of Au surface oxidation processes, which previous literature associates with the formation of molecular O_2_ [16,17]. The surface remains oxidized in the presence of N_2_, and no strain variation is detected. As the oxidized Au surface is exposed to CO, chemisorbed O atoms are removed from the surface as a consequence of the oxidation of CO to carbon dioxide (CO_2_) [16,17]. Correspondingly, the NP Au foam recovers its initial volume. Again, subsequent exposure to N_2_ does not induce any strain variation.

Although the effect is almost undetectable in a single cycle, the repeated alternate exposure to the different gaseous environments results for the NP Au foam in irreversible strain. The data plotted in Figure 3b indicate that the strain response exhibits hysteresis in the long term. This suggests the occurrence of irreversible atomic rearrangements even in the elastic deformation range. Nevertheless, SEM does not reveal any significant modification of the NP Au structure. Accordingly, NP Au samples subjected and not subjected to cyclic exposure to different gaseous environments have comparable structures.

**Figure 4:**
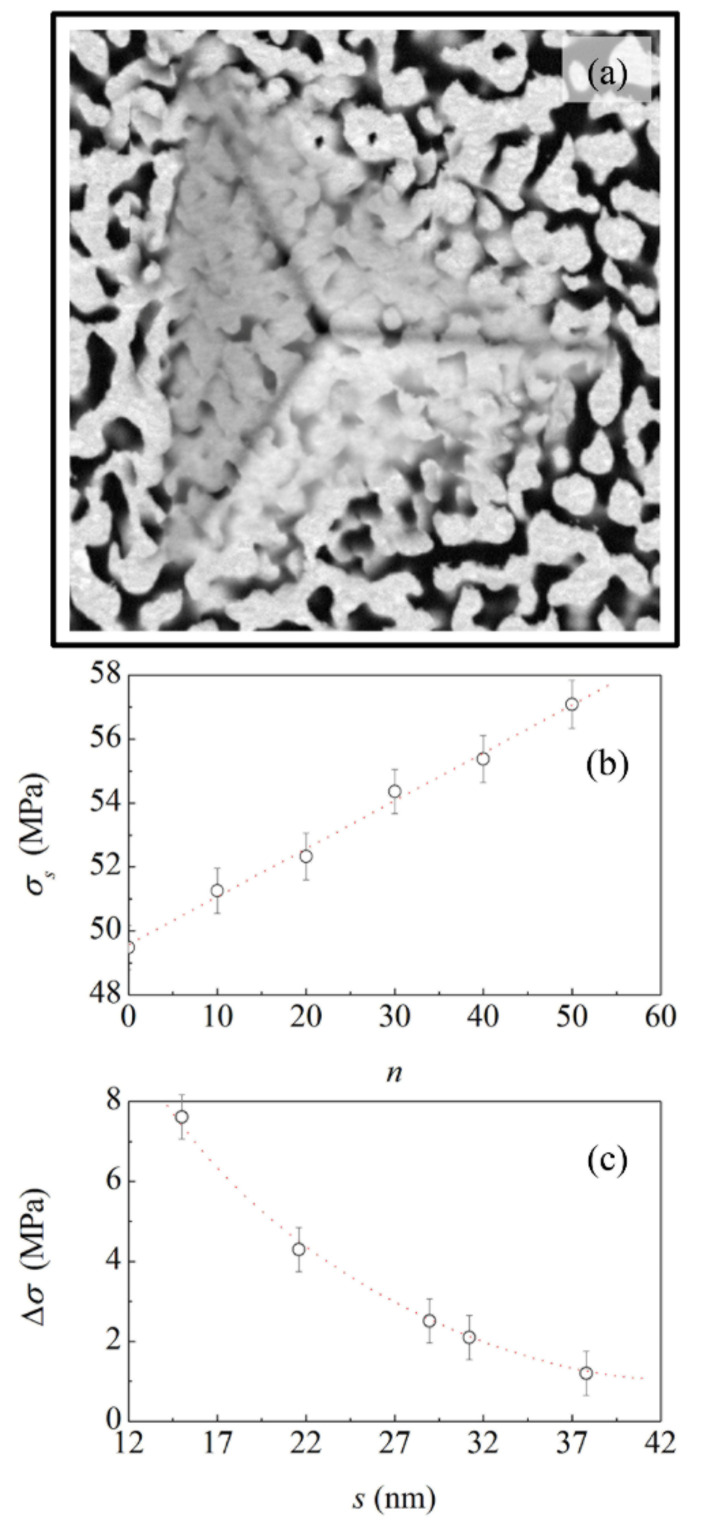
(**a**) SEM micrograph of the indentation area. The image refers to the NP Au structure annealed for 2 h. (**b**) The yield strength, as a function of the number of exposure cycles, *n*. Best-fitted line is shown. (**c**) The yield strength increase, Δ*σ*, as a function of the average ligament thickness, *s*. The curve is a visual guide.

In contrast, differences emerge in nanoindentation experiments. In agreement with the literature, the nanoindentation behaviour indicates that localized plastic densification is the dominant deformation mechanism for ductile NP Au. The SEM micrograph shown in Figure 4a and similar ones for the same and other samples clearly show that indentation induces the plastic deformation of the material directly and indirectly involved in the indentation area. Ligaments and nodes deform under mechanical loading, change mutual orientation and morphology, and are progressively compressed against each other, eventually leading to the localized compaction of the material. In contrast with the localized densification of compressed area, the surrounding regions remain substantially unaffected. It follows that NP Au is unable to propagate significant stress fields from a long range.

The yield strength, *σ_s_* of the NP Au foams subjected to cyclic exposure varies with the number of exposure cycles, *n*. The data shown in Figure 4b indicate an approximately linear increase with *n*. Starting from the initial value of about 49 MPa, which measures the yield strength, *σ_s_*, of the NP Au foam with ligaments about 15 nm thick, a value of about 57 MPa is reached after 50 exposure cycles. Thus, the cyclic exposure to the different gaseous environments finally results in a strengthening of the NP Au foam highlighted by an increase in yield strength, Δ*σ*, equal to about 8 MPa.

The available data do not allow for a proper resolution of the curve describing the smooth decrease with the ligament thickness. Overall, the experimental points can be hardly interpolated by a straight line. However, the paucity of data does not allow for concluding whether the experimental points can be best-fitted, for instance, by a power law or by an exponential function.

## 4. Conclusions

In summary, we performed a systematic investigation on the progressive strain hardening of NP Au foams induced by the cyclic exposure to O_3_ in O_2_ and CO gaseous environments. NP Au samples were fabricated using a conventional dealloying method in acidic solution. Parent alloys with the Ag_70_Au_30_ composition were prepared by mechanical alloying of elemental powders. The alloy powders were, then, compacted by cold pressing into thin cylinders. Cylindrical samples were immersed in an acidic solution of a suitable concentration for a long enough duration to obtain fine NP Au structures with ligaments about 15 nm thick. Pristine NP Au structures were annealed for different time intervals at 473 K in order to obtain NP Au samples with increasingly thicker ligaments, up to about 40 nm. Finally, the different NP Au structures were exposed to the different gaseous environments. Regardless of the fineness of the NP structure, all samples investigated underwent a cyclic strain variation caused by the succession of surface oxidation and reduction processes due to the interaction of the NP Au surface with oxidizing and reducing gaseous atmospheres. The cyclic strain behaviour is invariably associated with irreversible strain contributions. In the case of the NP Au structure with ligaments about 15 nm thick, 50 exposure cycles to the different gases induce an increase in yield strength of about 14%. The strengthening behaviour is clearly affected by the ligament thickness. In particular, the increase in yield strength induced by cyclic exposure becomes progressively less pronounced as the ligament thickness increases.

The observed strain hardening behaviour allows for fine tuning of the yield strength of NP Au foams, and, in principle, of other NP metals. While it poses fundamental questions regarding the mechanisms responsible for strengthening on the nanometre scale, it also shows promise for applications in specific areas of science and engineering.

## Data Availability

Not applicable.
